# Molecular Profiling of KRAS, NRAS, and BRAF in Colorectal Cancer: Insights From a Libyan Cohort

**DOI:** 10.7759/cureus.97476

**Published:** 2025-11-21

**Authors:** Hoda M Tawel, Monira A Rahuma, Nada A Hweissa

**Affiliations:** 1 Department of Pathology, Faculty of Medicine, University of Zawia, Zawia, LBY; 2 Department of Public Health, Faculty of Biotechnology, University of Zawia, Zawia, LBY

**Keywords:** braf v600 mutation, colorectal cancer, kras g12c mutation, kras mutation, molecular pathology, nras mutation, personalized medicine

## Abstract

Introduction

Colorectal cancer (CRC) is a significant global health issue with a rising incidence, particularly high in Libya, posing serious public health concerns. This study aimed to determine the prevalence and clinicopathological associations of KRAS, NRAS, and BRAF mutations in a Libyan CRC cohort, as these mutations are key drivers of tumorigenesis and predictors of resistance to anti-epidermal growth factor receptor therapy. Investigating the molecular profile of Libyan CRC patients is essential for advancing personalized medicine and improving clinical outcomes in this population.

Methods

In this retrospective study, a standardized data abstraction sheet was used to systematically retrieve data from patient medical records, pathology reports, and molecular biology laboratory reports. Nonparametric tests were applied to assess associations between age, gender, tumor site, stage, and histology. The study included approximately 72 histologically confirmed CRC cases analyzed for molecular profiling.

Results

Colon cancer accounted for 58 (80.6%) of all cases. Most tumors, 32 (44.4%), were located on the left side, while 28 (38.9%) were on the right side, and 11 (15.3%) were identified in the rectum. According to the UICC staging system, 23 (31.9%) cases were classified as stage II, 28 (38.9%) as stage III, and 16 (22.2%) as stage IV. Histologically, most cases (38; 52.8%) were moderately differentiated adenocarcinomas. KRAS mutations were detected in 39 (54.2%) of patients, predominantly at codon 12. BRAF and NRAS mutations were identified in four (5.6%) and two (2.8%) patients, respectively. Patients with KRAS mutations were significantly older than their wild-type (WT) counterparts (p < 0.001). Although the relationship between RAS mutation status and histopathological subtype was not statistically significant (p = 0.057), a trend was observed. Mucinous adenocarcinoma was more common in tumors with BRAF/NRAS mutations than in KRAS mutant or WT tumors. No significant associations were found with gender, tumor stage, tumor size, or tumor location.

Conclusions

KRAS mutations are prevalent in Libyan CRC patients, especially in older adults, highlighting their clinical importance. The absence of a correlation with tumor stage in this cohort suggests RAS mutations may be pivotal in cancer initiation rather than progression. In patients with BRAF/NRAS mutations, the association with advanced age and a tendency toward mucinous histology provides valuable insight into local disease characteristics and supports efforts toward personalized therapy. Future studies with larger, prospective cohorts are needed to confirm these trends, incorporate prognostic data, and develop a more comprehensive molecular profile.

## Introduction

Colorectal cancer (CRC) is one of the most common cancers worldwide, ranking as the third most prevalent cancer in 2022, with an estimated 1.9 million new cases globally [1]. The global burden of CRC is projected to increase substantially, with a predicted 63% rise in new cases by 2040, reaching approximately 3.2 million cases [2]. In Libya, CRC is a significant public health concern, with incidence rates comparable to those observed in European countries. According to the National Cancer Registry report for 2020, CRC accounted for 16.3% of all cancers in Libya, representing 19.8% of cancers in males and 13.6% in females [3]. Additionally, a recent publication reported that CRC in western Libya affected 113 (55.9%) males and 89 (44.1%) females in the studied sample [4]. The age-standardized incidence rates were 27.4 per 100,000 for males and 24.8 per 100,000 for females [3]. These rates are notably higher than those in neighboring countries, indicating that Libya has one of the highest CRC incidence levels in the Northern Africa and Middle East region [5].

CRC is influenced by a combination of modifiable and non-modifiable risk factors. Diets high in red and processed meats, low in fiber, and sedentary lifestyles increase CRC risk. Obesity, smoking, and excessive alcohol consumption are also key contributors [6,7]. Non-modifiable risk factors include age, family history, and hereditary syndromes such as Lynch syndrome. Genetic mutations and ethnicity further influence risk, with men and certain populations being more affected [8].

CRC develops from the epithelial cells lining the colon or rectum and often progresses through the accumulation of genetic alterations over time. Among the most frequently observed oncogenic alterations in CRC are mutations in the RAS gene family, particularly KRAS, and, less commonly, NRAS [9,10]. These RAS genes encode small GTPases that function as essential signal transducers in the RAS/MAPK pathway, a signaling cascade regulating key cellular processes, including growth, differentiation, proliferation, survival, and apoptosis [9].

In normal cells, RAS proteins cycle between an inactive GDP-bound state and an active GTP-bound state, mediated by guanine nucleotide exchange factors and GTPase-activating proteins [11]. Activating RAS mutations, most commonly missense mutations at codons 12, 13, 59, 61, 117, and 146, impairs the intrinsic GTPase activity of RAS proteins, preventing their return to the inactive GDP-bound state [9,12]. This leads to constitutive activation of the downstream MAPK pathway, resulting in sustained signaling that promotes uncontrolled cell growth, proliferation, and survival, ultimately contributing to tumorigenesis and disease progression.

KRAS mutations occur in approximately 30-40% of CRC cases, making it one of the most frequently mutated oncogenes in this malignancy [13]. NRAS mutations are less common, occurring in about 3-8% of cases [14]. One of the most prevalent KRAS alterations occurs at codon 12, with substitutions such as G12D, G12V, G12C, G12S, and G12A replacing glycine with other amino acids, resulting in constitutive protein activation. Mutations at codon 13, particularly G13D, similarly compromise normal GTPase function and enhance oncogenic signaling [15]. Although less frequent, codon 61 mutations (including Q61H, Q61L, and Q61R) are clinically significant due to their destabilizing effects on the GTP-binding domain, severely impairing hydrolytic activity [15].

RAS mutations not only contribute to tumorigenesis but also serve as key predictive biomarkers for response to anti-epidermal growth factor receptor (EGFR) therapies such as cetuximab and panitumumab [16]. Patients with RAS-mutated CRC generally do not respond to these targeted agents, emphasizing the importance of RAS mutation testing in treatment selection [16]. Emerging evidence also suggests that specific RAS mutations may carry prognostic value, influencing disease-free and overall survival [13,17,18].

The objective of this study is to examine the incidence of KRAS gene mutations in a cohort of Libyan CRC patients and to correlate mutational status with key clinicopathological variables, including histological type, tumor stage, and patient demographics. The findings of this study are essential for supporting personalized medicine in Libya, optimizing treatment strategies, and improving patient outcomes.

## Materials and methods

Study setting and population

This retrospective, single-center, descriptive, and analytical study was conducted at the National Cancer Institute in Sabratha. The study population included all patients with a histologically confirmed diagnosis of colorectal adenocarcinoma whose records were archived at the institute and who underwent KRAS/BRAF gene testing between January 1, 2019, and December 31, 2020. Cases with incomplete data or unconfirmed diagnoses were excluded.

Data collection

Data were systematically retrieved from patient medical records, pathology reports, and molecular biology laboratory reports using a standardized data abstraction sheet. The collected variables included demographics (age at diagnosis, gender, and city/region of residence), clinical details (date of diagnosis and primary tumor location), and pathological characteristics (histopathological type, tumor grade, and pathological stage). Molecular data were obtained from existing reports on KRAS mutation status (wild-type (WT) or mutated, with specific mutation subtypes such as G12D or G13D) and BRAF V600E mutation status, using fully automated Biocartis Idylla™ real-time PCR technology (Biocartis NV, Mechelen, Belgium).

Data management and analysis

All statistical analyses were performed using IBM SPSS Statistics for Windows, Version 26.0 (Released 2018; IBM Corp., Armonk, NY, USA). Descriptive statistics for categorical variables were reported as frequencies and percentages. The distribution of RAS mutation subtypes (WT, KRAS, and other (BRAF/NRAS)) was examined within the cohort. The chi-square test of independence was used to assess relationships between nominal variables (e.g., gender, tumor site, organ, and histological subtype) and RAS mutation status. Fisher’s exact test was applied when expected cell counts were not met (i.e., more than 20% of cells had an expected count of fewer than 5).

The Kruskal-Wallis H test was used to evaluate associations between ordinal variables (e.g., cancer stage, age group, and tumor size categories) and RAS mutation status. When the Kruskal-Wallis test indicated statistical significance, Mann-Whitney U tests were used for post hoc pairwise comparisons. A Bonferroni correction was applied to adjust for Type I error due to multiple comparisons, resulting in an adjusted significance threshold of p < 0.0167 for paired tests. Statistical significance for all other analyses was defined as a two-tailed p-value < 0.05.

Ethical considerations

The National Cancer Institute in Sabratha officially authorized this study. Ethical approval was also obtained from the Libyan Medical Research Center's biosafety and bioethics committee (approval number NBC:018.H.24.60). Patient confidentiality was strictly maintained throughout the study, and results were reported in aggregate form to prevent identification of individuals.

## Results

Distribution of the demographic data of the tumor

A total of 72 histologically confirmed CRC cases were included in the study. These cases were recorded in the cancer registry of the Pathology Department at the National Institute of Oncology/Sabrata between 2019 and 2020 and were referred for further assessment of KRAS/BRAF mutations at the department’s molecular unit. The interquartile age range of the study population was 28-85 years, with a median age of 58 years. The largest age group was 51-60 years, comprising 25 (34.7%) of the study population. Gender distribution was equal, with 36 (50%) males and 36 (50%) females.

Colon cancer accounted for 58 (80.6%) of all cases. Most tumors, 32 (44.4%), were located on the left side, while 28 (38.9%) were on the right side, and 11 (15.3%) were identified in the rectum. Table 1 summarizes the demographic characteristics of the analyzed cases.

**Table 1:** Demographic features of the study population (n = 72)

Variable	Category	N (%)
Age group	Younger than 20	0 (0.0%)
	21-30	3 (4.2%)
	31-40	3 (4.2%)
	41-50	14 (19.4%)
	51-60	25 (34.7%)
	61-70	15 (20.8%)
	Older than 70	12 (16.7%)
Gender	Male	36 (50.0%)
	Female	36 (50.0%)
Tumor site	Colon	58 (80.6%)
	Colo-rectum	2 (2.8%)
	Rectum	11 (15.3%)
	Other	1 (1.4%)
Tumor location	Left	32 (44.4%)
	Right	28 (38.9%)
	Whole colon	1 (1.4%)
	Rectum	11 (15.3%)

Distribution of tumor sizes, tumor grades, UICC stages, and T & N classifications

The distribution of tumors according to T and N classifications, UICC stage, and tumor grade is presented in Table 1. Most cancers, 46 (63.9%), had spread to the outermost layers of the colon or rectum but had not penetrated them (T3). Positive lymph nodes were identified in 45 (62.5%) of the cases. According to the UICC staging system, 23 (31.9%) cases were classified as stage II, 28 (38.9%) as stage III, and 16 (22.2%) as stage IV. Histologically, the majority of cases, 38 (52.8%), were moderately differentiated adenocarcinomas.

Overall, the median tumor size was 13.4 cm² (mean: 19.3 cm²). Among the tumors, 34 (47.2%) measured more than 8 cm². The distribution of tumor sizes is shown in Table 2.

**Table 2:** T and N classification, UICC stage, and tumor grading of CRC cases (n = 72) CRC, colorectal cancer

Variable	Category	N (%)
Tumor size (cm²)	<1	23 (31.9%)
	1.1-2	1 (1.4%)
	2.1-3	1 (1.4%)
	3.1-4	3 (4.2%)
	4.1-5	2 (2.8%)
	5.1-6	2 (2.8%)
	6.1-7	4 (5.6%)
	7.1-8	2 (2.8%)
	>8	34 (47.2%)
Histopathological grading	Well-differentiated adenocarcinoma	17 (23.6%)
	Moderately differentiated adenocarcinoma	38 (52.8%)
	Poorly differentiated adenocarcinoma	2 (2.8%)
	Mucinous adenocarcinoma	10 (13.9%)
T classification	T1	0 (0.0%)
	T2	6 (8.3%)
	T3	46 (63.9%)
	T4	20 (27.8%)
N classification	N0	27 (37.5%)
	N1	22 (30.6%)
	N2	23 (31.9%)
UICC stage	Stage 0	0 (0.0%)
	Stage I	5 (6.9%)
	Stage II	23 (31.9%)
	Stage III	28 (38.9%)
	Stage IV	16 (22.2%)

Prevalence of KRAS/BRAF and NRAS mutations in CRC

**Figure 1:**
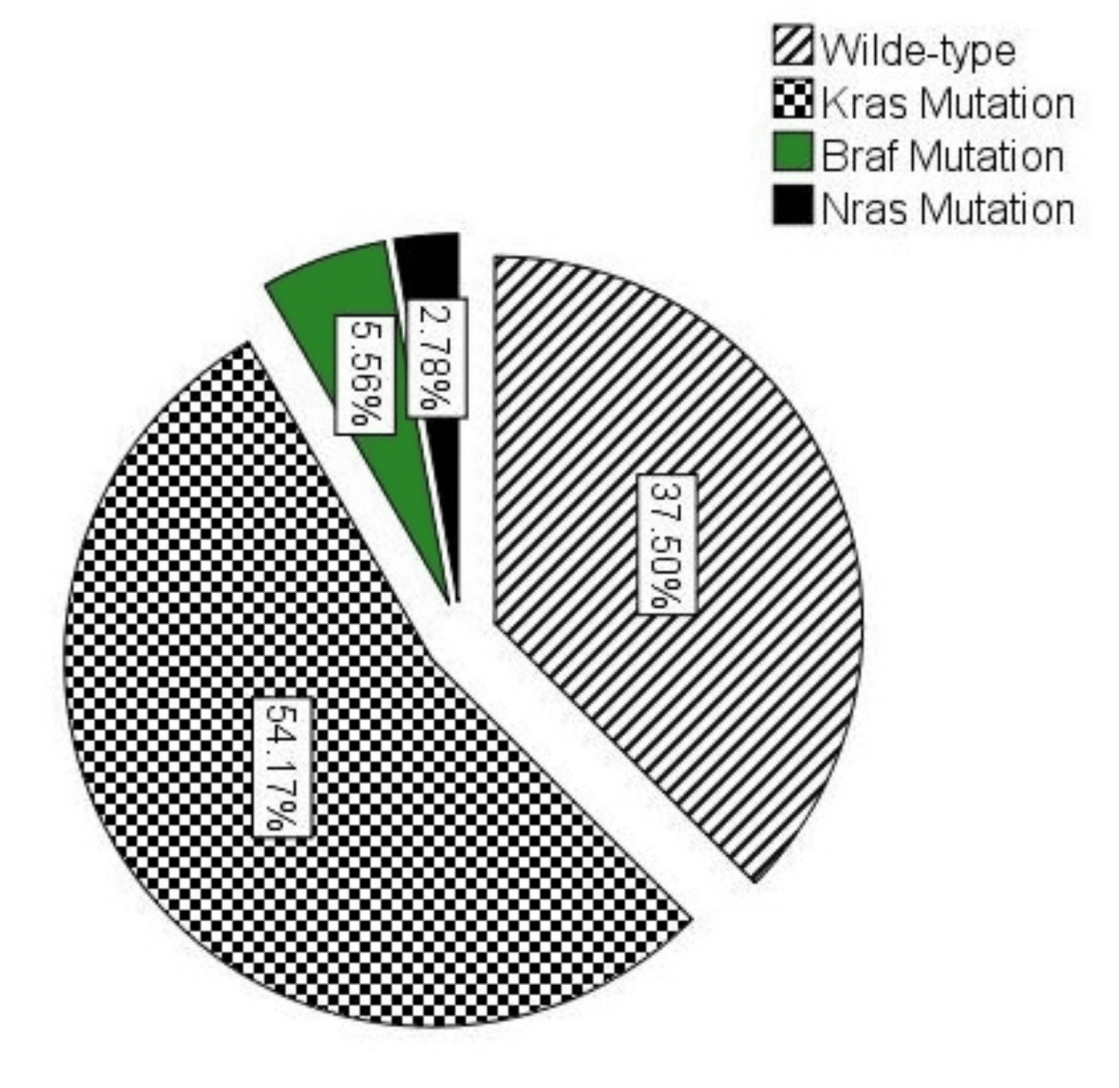
Frequency of KRAS, BRAF, NRAS, and HRAS mutations in the examined biopsies (n = 72) The majority of tumors harbored KRAS mutations, detected in 39 (54.17%) of the cases. WT RAS was observed in 27 (37.50%) cases. BRAF and NRAS mutations were considerably less common, occurring in four (5.56%) and two (2.78%) cases, respectively. WT, wild-type

In the evaluated samples, KRAS mutations were identified in 39 (54.2%) of the cases, while BRAF and NRAS mutations were detected in four (5.6%) and two (2.78%) cases, respectively. The remaining 27 (37.5%) samples showed the WT HRAS gene (Figure 1). The most prevalent alteration among KRAS mutations occurred at codon 12, accounting for 31 (43.1%) of all cases. In contrast, mutations at codon 13 and codon 146 were less common, identified in five (6.9%) and three (4.2%) tumors, respectively. Overall, BRAF and NRAS mutations were substantially less frequent than KRAS mutations, as shown in Table 3.

**Table 3:** Frequency of codon changes in mutated KRAS/BRAF/NRAS genes (n = 72) WT, wild-type

Mutation type	Codon/variant	Cases, N (%)
Human WT RAS	-	27 (37.5%)
KRAS mutation	Codon 12	31 (43.1%)
	Codon 13	5 (6.9%)
	Codon 146	3 (4.2%)
BRAF mutation	Codon 600	4 (5.6%)
NRAS mutation	Codon 61	1 (1.4%)
	Codon 117	1 (1.4%)
Total	-	72 (100%)

The mutational profile of KRAS, presented in Table 4, demonstrated distinct patterns of oncogenic alterations. The most common mutation in the KRAS-mutated group (n = 39) was the c.34G>T variant at codon 12, detected in nine (23.1%) cases. This variant is associated with constitutive activation of the MAPK and PI3K signaling pathways, thereby promoting neoplastic proliferation. The c.35G>T variant at codon 12 was observed in eight (20.5%) cases and is clinically significant due to its association with aggressive tumor behavior and reduced survival in colorectal carcinoma. Another pathogenic variant of prognostic importance, c.35G>A, was found in seven (17.9%) cases. Mutations at codon 13, particularly c.38G>A, were identified in five (12.8%) specimens. Codon 146 mutations, which exhibit moderate oncogenic potential (c.436G>C/A and c.437C>T), were less frequent, occurring in three (7.7%) cases.

**Table 4:** Prevalence of KRAS mutations according to nucleotide changes (n = 39)

RAS mutation		Amino acid changes	N (%)	Cumulative, %
Codon 12	c.34G>A	p.Gly12Ser	3 (7.7%)	7.7
	c.34G>C	p.Gly12Arg	2 (5.1%)	12.8
	c.34G>T	p.Gly12Cys	9 (23.1%)	35.9
	c.35G>A	p.Gly12Asp	7 (17.9%)	53.8
	c.35G>C	p.Gly12Ala	2 (5.1%)	59
	c.35G>T	p.Gly12Val	8 (20.5%)	79.5
Codon 13	c.38G>A	p.Gly13Asp	5 (12.8%)	92.3
Codon 146	c.436G>C/A; c.437C>T	p.Gly146Arg; p.Gly146Ser; p.Gly146Gly	3 (7.7%)	100
Total	-	-	39 (100%)	-

Relationships between clinicopathological variables

Correlation analyses were performed to examine associations between clinicopathological features in 72 tumor samples. First, the relationship between tumor size and other variables (age group, UICC stage, and T/N stage) was evaluated using Spearman’s rank correlation. Significant correlations were observed between tumor size and tumor staging (both UICC and T/N stages) (p < 0.001), suggesting interrelated pathological progression patterns. However, no significant association was found between patient age and either tumor size or tumor stage (Table 5).

**Table 5:** Spearman’s Rho correlation analysis between tumor size, tumor stages (UICC and T/N stages), and age groups (n = 72) ^**^ Correlation is significant at the 0.01 level (two tailed).

Variable	Tumor size	Age group	UICC stage	T stage	N stage
Tumor size	1	0.018	0.437^**^	0.323^**^	0.456^**^
Sig. (two tailed)	-	0.884	0	0.006	0
N	72	72	72	72	72
Age group	0.018	1	0.007	-0.073	-0.085
Sig. (two tailed)	0.884	-	0.952	0.54	0.477
N	72	72	72	72	72
UICC stage	0.437^**^	0.007	1	0.503^**^	0.815^**^
Sig. (two tailed)	0	0.952	-	0	0
N	72	72	72	72	72
T stage	0.323^**^	-0.073	0.503^**^	1	0.586^**^
Sig. (two tailed)	0.006	0.54	0	-	0
N	72	72	72	72	72
N stage	0.456^**^	-0.085	0.815^**^	0.586^**^	1
Sig. (two tailed)	0	0.477	0	0	-
N	72	72	72	72	72

Additionally, no significant associations were observed between histopathological tumor subtype and gender, tumor size, or tumor location using the chi-square test (data not shown). A Mann-Whitney U test revealed no statistically significant difference in tumor stage between males (mean rank = 38.31) and females (mean rank = 34.69), U = 583, p = 0.44. Similarly, tumor location (left vs. right) did not differ significantly between genders, U = 564, p = 0.30.

Correlation between KRAS mutation and clinicopathological characteristics

To evaluate the relationship between RAS mutations and clinicopathological characteristics, patients were categorized by KRAS, BRAF/NRAS, or WT status. Associations between RAS mutation status and ordinal factors (tumor stage, age group, and tumor size) were assessed using the Kruskal-Wallis H test. A significant difference in age group distribution was observed across RAS mutation types (χ²(2, N = 72) = 13.406, p < 0.001), indicating that patient age profiles varied according to mutational status. However, no significant associations were found between mutation status and tumor stage (χ²(2, N = 72) = 1.36, p = 0.50) or tumor size (χ²(2, N = 72) = 0.034, p = 0.98).

Post hoc pairwise analysis using Mann-Whitney U tests with a Bonferroni-adjusted alpha level of 0.0167 revealed the underlying pattern driving this significance. Patients with KRAS mutations (mean rank = 42.5) were significantly older than those with WT tumors (mean rank = 25.0) (U = 272, p = 0.001). After adjustment for multiple comparisons, no statistically significant differences were observed in age distribution between the BRAF/NRAS group (mean rank = 47.5) and the WT group (U = 34, p = 0.026), or between the BRAF/NRAS and KRAS groups (U = 97, p = 0.48).

**Figure 2:**
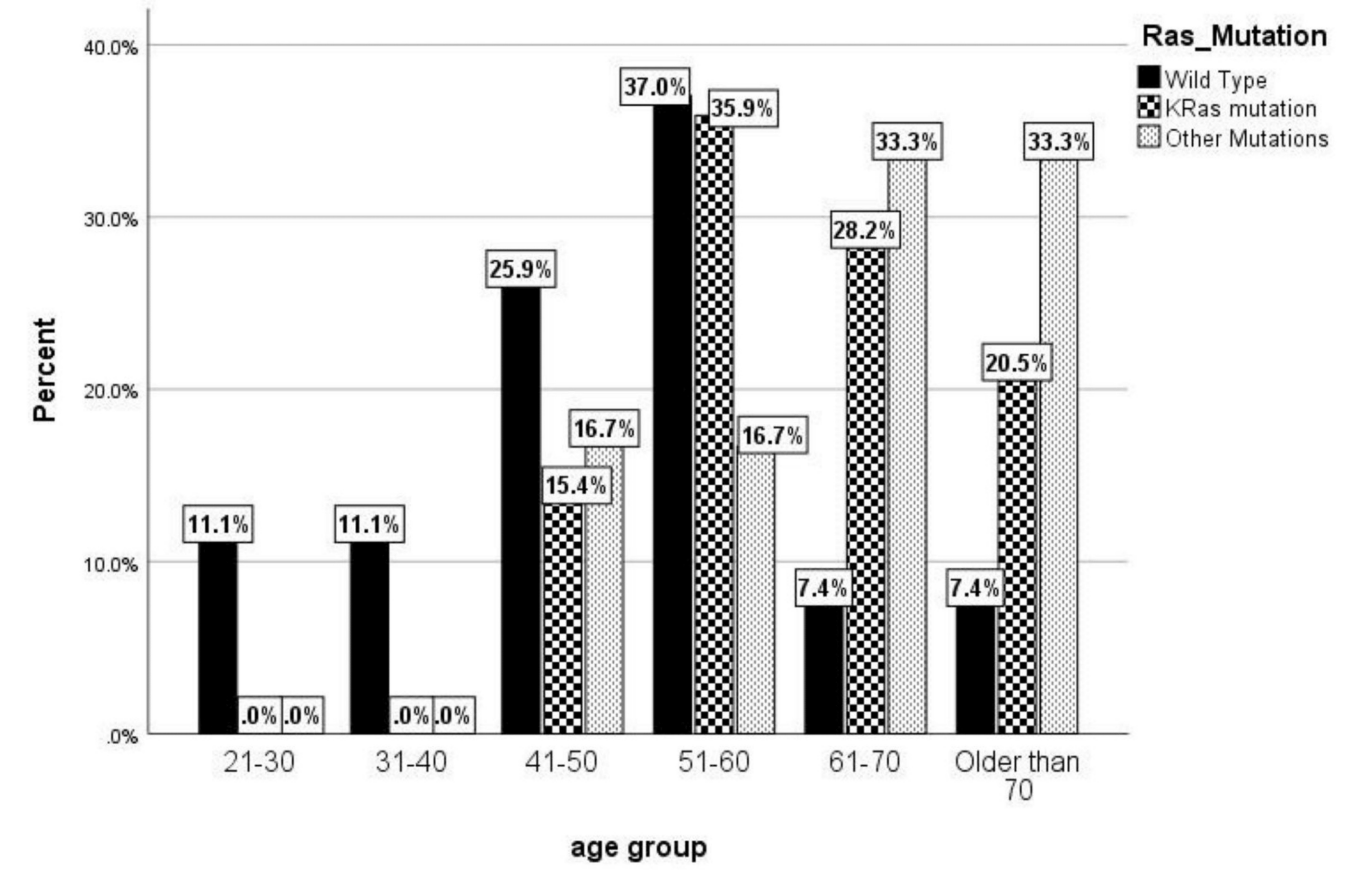
KRAS mutations across different patient age groups (n = 72) BRAF/NRAS mutations are more frequent in older patients (>61 years) compared with WT tumors, which are more common in younger patients. KRAS mutations are most prevalent in patients aged 51-71 years, contributing substantially to the overall age distribution. WT, wild-type

These findings suggest that the overall age difference is primarily driven by older age in patients with KRAS-mutated tumors compared with younger patients with WT tumors. Although the BRAF/NRAS group tended to be older, post hoc testing confirmed that their age distribution was not significantly different from the other groups (Figure 2).

There were no significant associations between RAS mutation groups and major demographic or anatomical factors. Fisher’s exact tests indicated that mutation status was not influenced by patient gender (p = 0.34), tumor site (rectum, colon, colorectum, or other) (p = 0.77), or tumor laterality (right vs. left) (p = 0.70).

Although the association between RAS mutation status and histopathological subtype was not statistically significant (p = 0.057), a trend was observed. Mucinous adenocarcinoma was more common in tumors with BRAF/NRAS mutations (two of six cases, 33.3%) compared with KRAS-mutated tumors (six of 39 cases, 15.4%) or WT tumors (two of 27 cases, 7.4%). In contrast, mixed well-to-poorly differentiated adenocarcinomas were less frequent in the BRAF/NRAS group (three of six cases, 50.0%) than in the KRAS (33 of 39 cases, 84.6%) or WT (21 of 27 cases, 77.7%) groups (Table 6).

**Table 6:** RAS mutation status across histopathological subtypes of colorectal tumors (n = 72) Values are presented as column percentages. p-Value from Fisher’s exact test = 0.057. WT, wild-type

Histopathological subtype	WT (n = 27)	KRAS mutation (n = 39)	Other mutations (n = 6)
Well-differentiated adenocarcinoma	6 (22.2%)	10 (25.6%)	1 (16.7%)
Moderately differentiated adenocarcinoma	13 (48.1%)	23 (59.0%)	2 (33.3%)
Poorly differentiated adenocarcinoma	2 (7.4%)	0 (0%)	0 (0%)
Mucinous adenocarcinoma	2 (7.4%)	6 (15.4%)	2 (33.3%)
Other subtypes	4 (14.8%)	0 (0%)	1 (16.7%)
Total conventional adenocarcinoma	21 (77.7%)	33 (84.6%)	3 (50.0%)
Total non-conventional subtypes	6 (22.2%)	6 (15.4%)	3 (50.0%)

These findings suggest a potential biological predisposition for BRAF/NRAS-mutated tumors to develop a mucinous phenotype, which warrants further investigation in a larger study cohort. 

## Discussion

This study provides an in-depth analysis of the prevalence of RAS mutations and their clinicopathological associations in a cohort of Libyan CRC patients. The results underscore the importance of KRAS mutations in colorectal carcinogenesis and treatment resistance. Notable correlations between tumor characteristics, such as tumor size and stage, were observed, suggesting synergistic patterns in tumor development. Key findings include a high frequency of KRAS mutations, a trend linking BRAF/NRAS mutations to mucinous adenocarcinoma, and a significant association between KRAS mutations and older patient age. These findings have implications for personalized therapeutic strategies in Libya and contribute valuable population-specific data to the global understanding of CRC etiology.

The prevalence of KRAS mutations in our cohort, 39 (54.2%), was substantially higher than the global average of 30-40% [19] and higher than previously reported in eastern Libya (38.3%) [12]. This elevated prevalence may reflect geographic variation in genetic predisposition, environmental exposures, or lifestyle factors influencing carcinogenesis, and is consistent with studies in other Middle Eastern and North African populations [9,12]. Consistent with prior research, codon 12 mutations were the most frequent, particularly c.34G>T (p.Gly12Cys) and c.35G>T (p.Gly12Val), which stabilize the GTP-bound KRAS protein and drive constitutive MAPK pathway signaling, marking this codon as a critical hotspot for oncogenic activation [9,12,15].

A key finding of this study is the significant association between RAS mutation status and patient age. Patients with KRAS-mutated tumors were significantly older than those with WT tumors (p = 0.001). This observation aligns with carcinogenesis models in which KRAS-mutated cancers often develop through a slower, cumulative pathway. In this model, KRAS mutations act as initiating events, requiring a longer latency and the accumulation of additional genetic alterations before clinical cancer develops [20]. In contrast, some WT tumors may arise through more aggressive pathways, such as microsatellite instability (MSI) or hypermethylation, and present at a younger age. Age-related declines in DNA repair mechanisms and lifelong exposure to dietary mutagens may further favor the acquisition and clonal expansion of KRAS-mutated cells [21].

Although formal statistical significance was not achieved, likely due to the small sample size of the BRAF/NRAS group, a notable trend was observed between these mutations and histology. Mucinous adenocarcinoma occurred more than twice as frequently in BRAF/NRAS-mutated tumors as in KRAS-mutated tumors, and more than four times as frequently as in WT tumors. This supports a biological link between BRAF V600E mutations and the serrated pathway of carcinogenesis, which is associated with CpG island methylator phenotype (CIMP), right-sided tumor location, and mucinous differentiation [19,22]. NRAS mutations, though less frequent, may share similar oncogenic mechanisms, highlighting the need for further investigation.

Unexpectedly, no significant associations were found between RAS mutation status and other clinicopathological characteristics, including tumor stage, size, gender, or anatomical location. The lack of correlation with advanced stage (TNM or UICC) suggests that in this Libyan population, RAS mutations may be more important for tumor initiation than for determining progression or metastatic potential. This contrasts with Western studies linking specific KRAS mutations to more advanced disease and poorer survival [17,23], suggesting potential population-specific differences in cancer behavior. The absence of an association with tumor location (left vs. right) also differs from the well-established connection between BRAF mutations and right-sided tumors [24], which may reflect the small number of BRAF-mutant cases in this cohort or unique etiological factors. Larger studies are needed to explore this further.

The high prevalence of KRAS mutations in Libyan CRC patients underscores the clinical importance of routine genetic testing. Since over 50% of patients harbor KRAS mutations, many would not benefit from anti-EGFR therapies such as cetuximab or panitumumab [16,25,26]. Integrating RAS testing into the national standard of care could prevent ineffective treatment, reduce morbidity, and lower healthcare costs. Moreover, the observed trend toward BRAF mutations and mucinous histology is clinically relevant, as BRAF-mutant CRC is associated with poor prognosis and may respond to targeted regimens combining BRAF/MEK inhibitors with anti-EGFR therapy [27].

Limitations and future directions

The statistical power of this study was limited by the small sample size (N = 72), which restricted the ability to perform robust multivariate analyses and detect associations with less common variants such as BRAF and NRAS. Selection bias may also have arisen from the retrospective, single-center design. Future research should involve larger, multi-center prospective cohorts with longitudinal follow-up to allow prognostic analyses and to more definitively validate the trends observed. Additionally, incorporating CIMP and MSI status would provide a more comprehensive molecular characterization and shed light on the underlying carcinogenic pathways in this population.

## Conclusions

KRAS mutations are highly prevalent in Libyan patients with CRC, particularly among older adults. Although not statistically significant, a notable trend linking BRAF/NRAS mutations to mucinous histology was observed and warrants further investigation. The lack of association between mutation status and tumor stage suggests that RAS mutations may play a greater role in tumor initiation than in progression in this cohort. Routine RAS mutation testing in Libya is essential for guiding personalized therapy, avoiding ineffective treatments, and improving patient outcomes.
